# Bright ideas: How leaf cells shape the way plants capture light

**DOI:** 10.1093/plphys/kiaf064

**Published:** 2025-03-04

**Authors:** Nicola Trozzi, Janlo M Robil

**Affiliations:** Assistant Features Editor, Plant Physiology, American Society of Plant Biologists; Department of Computational and Systems Biology, John Innes Centre, Norwich Research Park, Norwich NR4 7UH, UK; Department of Plant Molecular Biology, University of Lausanne, Lausanne CH-1015, Switzerland; Assistant Features Editor, Plant Physiology, American Society of Plant Biologists; Department of Biology, School of Science and Engineering, Ateneo de Manila University, Quezon City 1108, Philippines

Leaves are complex structures optimized to capture light and drive photosynthesis, a process that supports plant growth and forms the foundation of most ecosystems. At the heart of this efficiency lies the palisade mesophyll, a layer of cells located beneath the upper epidermis. These cells are primarily responsible for light absorption and carbon fixation, with their geometry playing a key role in these functions (Esau 1965; Terashima et al. 2011). Columnar-shaped palisade cells, common in many species, have been studied for their ability to guide light through the leaf and protect chloroplasts from photodamage (Kasahara et al. 2002; Tholen and Zhu 2011). However, alternative geometries, such as lobed palisade cells, are less understood despite their presence in a wide variety of plant taxa, including ferns (Chatelet et al. 2013; Trueba et al. 2022). The functional significance of these lobed forms remains an open question, particularly regarding their ability to adapt to different light environments or optimize photosynthetic processes. This gap in understanding highlights the need for studies that explore the relationship between cell shape, light interaction, and photosynthetic performance.

In this issue of *Plant Physiology*, Borsuk et al. (2024) explore how the geometry of palisade mesophyll cells influences light absorption and photosynthesis in 15 Viburnum species with differing palisade cell morphologies (Fig.). By examining both lobed and columnar cell forms, the study reveals distinct strategies for optimizing photosynthetic efficiency under varying light conditions. Using advanced imaging techniques, simulations, and physiological measurements, the authors demonstrate that while lobed cells adjust light absorption based on chloroplast positioning, columnar cells respond more strongly to light directionality. Remarkably, both cell types converge on similar photosynthetic productivity at the tissue level, emphasizing the versatility of plant adaptations to diverse environments.

**Figure.**
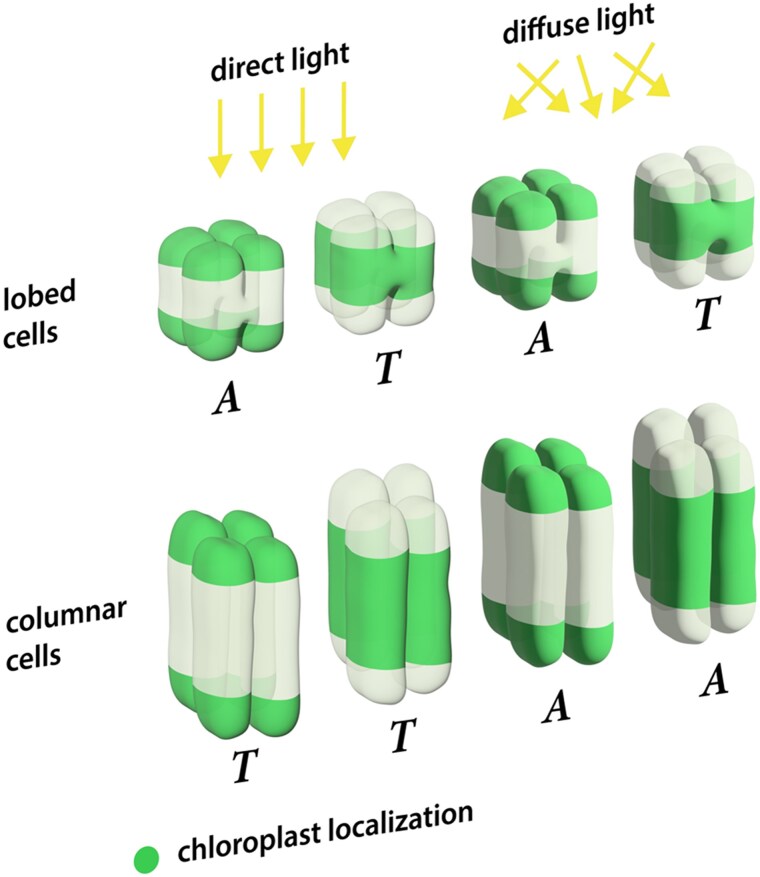
Adaptive light responses of lobed and columnar palisade cells in Viburnum. Schematic representation of lobed and columnar palisade cell geometries in Viburnum under direct and diffuse light. Lobed cells shift between absorptance (*A*) and transmittance (*T*) states based on chloroplast localization (green areas). Columnar cells, however, are responsive to light directionality, with increased absorption under diffuse light and greater transmission under direct light. Borsuk et al. (2024) illustrate how variation in cell geometries underpins distinct adaptations for maximizing photosynthetic efficiency in diverse environments.

Using micro-computed tomography, they reconstructed the 3-dimensional structures of palisade mesophyll cells in 15 Viburnum species, categorizing them into lobed or columnar forms. These data informed 3-dimensional ray tracing simulations, which allowed the researchers to model light interactions with individual cells and leaves under varying light conditions. The simulations revealed that lobed cells exhibit flexibility in light absorption, shifting between absorptance- and transmittance-dominated states depending on chloroplast localization. In contrast, columnar cells were more sensitive to light directionality, absorbing more light under diffuse conditions and transmitting more under direct light. These findings highlight the distinct strategies employed by each cell type to optimize photosynthetic efficiency in their specific light environments.

To validate their simulations, Borsuk et al. (2024) conducted in planta experiments measuring light absorption, reflectance, and transmittance in leaves from representative Viburnum species. The results confirmed that lobed cells, associated with species adapted to low-light or diffuse-light environments, absorbed light more evenly across different conditions. Conversely, columnar cells, often found in high-light environments, maximized absorption under direct light while allowing more transmission under diffuse light. The study further examined the movement of chloroplasts within these cells in response to weak and strong light conditions, revealing that chloroplast positioning significantly influenced the optical performance of lobed cells but had a lesser effect on columnar cells. The results closely matched the predictions from the ray tracing models, providing strong evidence for the relationship between cell geometry and optical responses to different light environments.

In addition to optical measurements, the study assessed photosynthetic performance by comparing gas exchange rates and maximum carbon assimilation among the species. While columnar cells had higher packing densities, lobed cells showed greater photosynthetic rates per individual cell, compensating for their lower density. This balance resulted in a convergence of productivity per unit of leaf tissue across both cell types. Notably, the lobed-cell increased surface area to volume ratio likely enhanced gas exchange efficiency, making them well-suited for low-light environments where maximizing resource capture is critical. The evidence suggests that the structural diversity in palisade cells represents a functional adaptation to specific light conditions rather than being solely a product of evolutionary variation.

The findings of Borsuk et al. (2024) demonstrate how variations in leaf anatomy, particularly in palisade cell geometry, enable plants to adapt to different light environments. The study highlights the distinct strategies different species use to optimize photosynthesis. Lobed cells adapt to diffuse light conditions, while columnar cells excel in direct sunlight. These findings offer valuable ecological insights into how plants respond to changing light environments, such as those driven by canopy dynamics or climate variability, depending on their palisade cell morphology. The research may have significant implications for agriculture, suggesting that tailoring leaf structures to specific light environments could enhance crop efficiency, whether in controlled settings or variable outdoor conditions.

Building on this work, future research could explore the relationship between leaf structure and overall plant performance in diverse environmental conditions. Understanding how palisade cell geometry influences not only light absorption but also water use efficiency and carbon capture could lead to the development of more resilient plants, better equipped to handle climate change. The study also raises important questions about the genetic and developmental mechanisms responsible for the formation of lobed versus columnar cells, which could provide opportunities for targeted genetic modifications. Additionally, future studies could examine how these anatomical traits interact with overall plant architecture, potentially improving plant productivity and sustainability in both natural and agricultural systems.

Borsuk et al. (2024) provide compelling evidence that the geometry of palisade mesophyll cells plays a critical role in optimizing light absorption and photosynthesis. By connecting cell shape to environmental light conditions, the study not only advances our understanding of leaf anatomy but also suggests strategies for improving plant productivity in various ecological and agricultural contexts.

## Data Availability

This study did not generate or analyze any new data.
